# Necessary Rules of Sleep

**Published:** 1888-04

**Authors:** Forbes Winslow


### NECESSARY RULES OF SLEEP.

There is no fact more clearly established in the physiology of man than this, that the brain expends its energies and itself during the hours of wakefulness, and that these are recuperated during sleep. If the recuperation does not equal the expenditure, the brain withers; this is insanity. Thus it is that in early English history, persons who were condemned to death by being prevented from sleeping, always died raving maniacs; thus it is, also, that those who are starved to death become insane,—the brain is not nourished, and they cannot sleep. The practical inferences are three: (1) those who think most, who do the most brain work, require the most sleep; (2) that time "saved" from necessary sleep is infallibly destructive to mind, body and estate; (3) give yourself, your children, your servants, give all that are under you, the fullest amount of sleep they will take, by compelling them to *go to bed* at some regular, early hour, and to rise in the morning the moment they awake; and within a fortnight, nature, with almost the regularity of the rising sun, will unclose the bonds of sleep the moment enough repose has been secured for the want of the system. This is the only safe and sufficient rule; and as to the question of how much sleep any one requires, each must be a rule for himself; great Nature will never fail to write it out to the observer under the regulations just given.—*Dr. Forbes Winslow.*

